# Quality Changes in Black Chokeberry Juice Treated by Thermal-Assisted High Hydrostatic Pressure during Cold Storage

**DOI:** 10.3390/molecules27185892

**Published:** 2022-09-11

**Authors:** Guoliang Jia, Minghao Jiang, AiDong Sun, Zhilin Gan

**Affiliations:** 1 Department of Food, College of Biological Sciences and Technology, Beijing Forestry University, Beijing 100083, China; 2 Beijing Key Laboratory of Forest Food Processing and Safety, Beijing Forestry University, Beijing 100083, China

**Keywords:** aronia juice, pasteurization, thermal-assisted high hydrostatic pressure, storage

## Abstract

The effects of thermal-assisted high hydrostatic pressure (TAHHP), high hydrostatic pressure (HHP), and thermal pasteurization (TP) treatments on the quality of aronia juice were evaluated in this study. The results showed that TAHHP and HHP significantly decreased the aerobic plate counts of aronia juice. No significant differences in terms of physicochemical properties, such as pH and total soluble solids, were observed between aronia juice treated with high pressure or thermal pasteurization treatment after 28 days of storage. TAHHP and HHP affected the colour and antioxidant characteristics of aronia juice, though to a significantly lower extent than TP. This result demonstrates that TAHHP and HHP can better maintain the original quality of aronia juice than TP. In summary, both TAHHP and HHP can maintain the microbiological safety and original quality characteristics of aronia juice. TAHHP can effectively increase the safety and duration of cold storage of aronia juice, and hence is highly useful for the juice industry.

## 1. Introduction

Aronia, known as chokeberry, is a member of the Rosaceae family, a native of Eastern North America. Black chokeberry (*Aronia melanocarpa*) is a species of Aronia that is cultivated commercially, principally in Eastern Europe, for use in the production of juice, jam, syrup, wine, and natural food colorants [1]. Aronia has a unique composition of bioactive compounds, including anthocyanins and procyanidins at concentrations as high as 2% and 5% for the berry dry matter, respectively [2]. However, flavanols have been found to constitute only 1.3% of the total phenolic compounds in aronia (7849.21 mg/100 g of dried matter) [3,4]. Black chokeberry is considered to be a source of compounds with a wide spectrum of health benefits [5,6]. However, fresh aronia is rarely consumed because of several negative sensory attributes, such as bitterness and astringency. Therefore, aronia is instead used to produce juices, wines, and anthocyanin colorants. Processing for juice production involves high-temperature treatment, such as pasteurization, which strongly diminishes product quality. Recent studies have shown that over 70% of consumers associate emerging nonthermal technologies with higher nutrient or sensory quality than thermal treatment [7].

High hydrostatic pressure (HHP) is an innovative processing technology wherein food is exposed to pressure (up to 600 MPa) for a short duration along with treatment at a specified temperature [8]. Several attempts have been made to use HHP instead of high temperatures to inactivate food-spoiling microorganisms and undesirable food enzymes while maintaining all the quality and safety parameters of the products [9]. HHP treatment can typically induce a sublethal injured state in microorganisms. High temperature can lead to further loss of viability of microorganisms under the same pressure conditions [10]. A synopsis of the mechanism of inactivation of microorganisms by HHP is that high pressure induces a series of physiological changes in microbial cells, e.g., changes in the microbial permeability (rupture of the cell structure), resulting in the loss of normal metabolic functions [11]. However, these sublethal microorganisms can be easily missed in routine detection and may recover during juice storage, corrupting the product and shortening its shelf life. It has been reported that mild heat can augment the efficacy of a pressure-based pasteurization treatment to a great extent [12]. Moreover, HHP treatment has positive impacts the on a variety of juices (e.g., mango, blueberries and grapefruit) [13]. Therefore, the aim of the present study was to determine the optimal processing parameters of thermal-assisted high hydrostatic pressure treatment (TAHHP) for reducing the content of sublethal microorganisms and to characterize the effect of this treatment on the microbiological stability, polyphenol content, antioxidant capacity, and other quality attributes of aronia juice during cold storage (4 °C).

## 2. Results

### 2.1. Changes in Microbial Counts in Juice

The optimal process for deastringency treatment of black chokeberry stock juice was found to be the addition of 18.5 mL water and 1.5 mL gelatine to every 10 mL of stock juice (temperature: 15 °C; time: 15 min). Here, the factors (gelatine content, temperature and time) were determined based on the deastringency results from an orthogonal experiment. The removal content of tannins was 30.9% under such conditions. An electronic nose was used to determine the decrease in the astringent taste and tart flavour of the juice, with the results being as high as 92.5% and 35.3%, respectively. The effects of TAHHP on microorganism levels in aronia juice were determined (Table 1). TAHHP remarkably reduced microorganism counts (*P* < 0.05) in juice. TAHHP at 200 MPa, 300 MPa, and 400 MPa led to a significant decrease in microbe numbers in juice. Overall, with increasing pressure (200–400 MPa), temperature (50–55 °C), and treatment time (2–10 min) there was an increasing tendency for the logarithmic reduction value of APC, indicating that more microbes were inhibited. No microorganisms were detected in juice treated by TAHHP at 400 MPa combined with thermal treatment at 50 °C. More intense HHP treatment may reduce the number of sublethal microorganisms and make them more sensitive to temperature.

Considering the aforementioned sterilization effects, storage experiments were carried out under 300 MPa and 400 MPa (Table 2). Among the 400 MPa treatments, the 400 MPa/7 min-50 °C/2 min treatment was not used based on the preliminary experimental results, while the single HHP (400 MPa/7 min) treatment was used as a control. Microorganism counts in all the samples were not detected at the beginning of storage, and only TAHHP at 300 MPa/7 min-55 °C/10 min and 400 MPa/7 min-50 °C/6 min could ensure that microorganisms were not detected in the samples after storage at 4 °C for 30 days; thus, these treatments prolonged the shelf-life of the juice. TAHHP can therefore be considered the optimal treatment among all of the considered treatments for increasing the storage time of aronia juice at low temperature. 

### 2.2. Antioxidant Characterization

The effects of TAHHP-55 (300 MPa/7 min-55 °C/10 min), TAHHP-50 (400 MPa/7 min-50 °C/6 min), HHP (400 MPa/7 min) and heat treatment (TP, 75 °C/30 s) on the antioxidant properties of aronia juice were investigated after 28 days of cold storage of the juice (Table 3).

As HHP resulted in tissue softening, the increase in the contents of the chemical components of HHP-treated tissues may be attributed to the destruction of plant cells and tissues, which resulted in the extraction of these chemicals. There was a decreasing tendency in the anthocyanin content of aronia juice as the storage duration increased. There were no significant differences in the procyanidin content among the treatment groups. Strong heat treatment (TP) had a significant effect on the content of anthocyanidins and no significant effect on the content of procyanidins. There were few differences for the content of total flavonoids among each treatment groups. While TP significantly affected the content of total phenolics, the total phenol content decreased with increasing storage duration, similar to the trend observed for anthocyanin substances. High-pressure assisted sterilization significantly affected the antioxidant capacity of juice, resulting in higher DPPH and ABTS scavenging activities after treatment. The results of the present study show that HHP has mildly destructive effects on the bioactive components of aronia juice, and can promote the retention of natural plant nutrients and the relatively high natural antioxidant ability for fruits and vegetables.

### 2.3. Physicochemical Characteristics

Table 4 shows the effects of TAHHP, HHP, and TP on the total soluble solids (°Brix), pH, and colour change (ΔE) of aronia juice after 28 days of storage at 4 °C. Neither the treatment type nor the storage time had a significant effect on the total soluble solids content of aronia juice (which was 6.1 ± 0.0 °Brix for the control group). By contrast, the pH value of the juices (the pH of the control group was 3.8 ± 0.0) decreased with time. Microorganisms metabolize the sugar in fruit juice to produce organic acids, thereby decreasing the pH, which may explain why the pH of the aronia juice was found to decrease in this study. The extent of the colour change of the juice increased with the processing temperature. Moreover, the ΔE value changed significantly with prolonged storage (*P* < 0.05). Treatment temperature has been found to significantly affect the colour of fruit juice. Nayak et al. reported that HHP preserved the physicochemical properties of apple juice, such as its pH, sugar content, total acidity, viscosity, and colour [14], while the aroma and flavour characteristics of HHP-treated fruit juice samples did not change during storage at 4 °C. HHP has been associated with higher natural colour retention than TP for blueberry puree [15].

The above-mentioned results show that the use of effective HHP methods to kill microorganisms in foodstuffs could help to preserve the quality of vegetable and fruit products during cold storage. TAHHP can effectively maintain the microbiological safety of foodstuffs with fewer negative effects on product quality parameters than other treatment methods.

## 3. Discussion

Astringency is caused by the binding of polyphenols (tannins) to salivary proteins, which causes the proteins to precipitate and aggregate, generating a dry and unpleasant sensation in the mouth. Consequently, the astringency intensity is determined by measuring the concentration of tannins [16]. The reaction between gelatine and polyphenolic substrates has been reported to inhibit protein–tannin precipitation, resulting in more than 20–22% astringency reduction [17]. The astringency reduction in this work reached as much as 30.9%.

Previous studies have shown that HHP can extend the shelf-life of plant-based foodstuffs. HHP application can effectively maintain the desired sensory attributes of untreated juice more effectively than TP treatment [18]. All of the aforementioned studies showed that HHP inhibits the growth of microorganisms in fruit and vegetable products during cold storage. In summary, TAHHP can enhance the sterilization effect and could provide an alternative to thermal processing. In this study, TAHHP at 300 MPa/7 min-55 °C/10 min and 400 MPa/7 min-50 °C/6 min was found to ensure microbiological safety for aronia juice during storage at 4 °C for 30 days, whereas HHP could not. An analysis of the effects of the treatments on the physicochemical properties of aronia juice showed that TAHHP and HHP caused significant colour changes in the juice, which were, however, less intense than those observed for TP-treated juices. In addition, fruit juices treated with TAHHP and HHP retained more antioxidants and had a higher antioxidant capacity than TP-treated juices. Therefore, compared to TP treatment, TAHHP treatment of aronia juice resulted in higher retention of quality characteristics (e.g., ΔE) as well as of natural nutritional compounds that are beneficial to human health. Chen et al. reported that treatment with HHP at 400 and 600 MPa for two treatment times (10 and 20 min) decreased the total mesophilic bacteria in asparagus juice to undetectable levels similarly to thermal treatment, with few changes in the pH and soluble solids contents, while maintaining significantly higher ascorbic acid, rutin, total phenolic contents, and total antioxidant activity for the juice compared to thermal treatment [19]. Alexandrakis et al. observed a slight increase in the antioxidant activity of sea buckthorn after HHP treatment [20]. A comparative analysis of juice treated with HHP (550 MPa, 5 min) and HTST (110 °C, 8.6 s) showed microbial safety and acceptable colour during 25 days of storage for both treatments, whereas HHP preserved the original flavour more effectively than HTST [21]. It was shown that combination of mild heat treatment with the HHP and other non-thermal technologies with different operating conditions can be highly effective for watermelon juice preservation [20]. These results show that HHP promotes extraction of antioxidants, preservation of functional components, and reduction of natural nutrient loss. As the cost of a nonthermal instrument is high, efforts should be made to develop a large-scale industrial sterilization device to implement TAHHP.

## 4. Materials and Methods

### 4.1. Materials and Chemicals

Black chokeberry fruit was collected in Dandong, Liaoning Province, and transported to the laboratory. The samples were stored in a refrigerated room (−20 °C) until juice preparation. All the chemicals (2,2-diphenyl-1-picrylhydrazyl (DPPH), 6-hydroxy2,5,7,8-tetramethylchroman-2-carboxylic acid (Trolox), and 2,2′-azinobis(3ethylbenzothiazoline-6-sulfonic acid) diammonium salt (ABTS)) used in this study were of analytical grade.

### 4.2. Preparation of Aronia Juice

The frozen samples were thawed in a 4 °C environment. Then, the fruit was blanched at 95 °C for 15 s. The samples were cooled in an ice water bath and mixed with distilled water (*w*/*w* = 1:2) to extract the juice, to which ascorbic acid (0.1%, *w*/*w*) was added for colour preservation. Pectinase (0.05%, *w*/*w*) was then added to the mixture at 45 °C and allowed to react for 2 h. Then, the samples were cooled to room temperature, centrifuged at 4000× *g* for 20 min, and filtered through four layers of cloth. The stock juice was stored for no longer than 3 days in the dark at 4 °C before being subjected to deastringency treatment.

To optimize the deastringency process, the content of soluble tannins was measured as the indicator using the methods described by Neumann et al., (2022) [22]. To measure the total polyphenols, the samples were reacted with phosphomolybdotungstic reagent in the dark and the absorbance of the resulting mixture was measured at 765 nm. A standard curve was generated using gallotannic acid. A stock solution of the juice was diluted ten-fold before being used for measurements. Astringency removal was realized by the addition of gelatine. An orthogonal experiment considering the temperature (5–25 °C), time (10–20 min), and gelatine addition (1–2 mL) was carried out to optimize the deastringency treatment, with an electronic tongue used to analyze changes in taste indicators.

### 4.3. Combination of High Hydrostatic Pressure (HHP) and Heat Treatment (HT)

In preparation for treatment, 20 mL of black chokeberry juice were transferred to an aseptic bag under aseptic conditions. The bag was sealed to exclude air. Samples were stored in a refrigerated room (4 °C) to await HHP (FB-110G5, Shanghai, China) and HT treatment.

Based on the preliminary experimental results, the juice samples were randomly assigned to different experimental groups and treated at 200, 300, and 400 MPa for 7 min and then at 45, 50, and 55 °C for 2, 6, and 10 min. For pasteurization, the 75 °C/30 s condition was realized by cooling the juice to room temperature using an ice-water bath. Storage experiments were performed on the samples for a period of 30 days at 4 °C. The samples were analyzed on Days 0, 10, 20, and 30 during storage.

### 4.4. Measurement of Total Microbial Counts

Microbiological counts for each sample were determined on Days 0, 10, 20, and 30 during storage to test the recovery and growth of microorganisms. Enumeration of aerobic bacteria, assessed as the aerobic plate count (APC), was performed after incubating the samples at 37 °C for 48 h. The microbial counts of experiments performed in triplicate are expressed as log CFU/mL.

### 4.5. Antioxidant Analysis

The total monomeric anthocyanins was determined using the pH differential method [23]. Briefly, 1 mL of a sample was mixed with 3 mL of KCl buffer (0.025 M) at pH 1.0 and 3 mL of CH_3_COONa buffer (0.4 M) at pH 4.5. The resulting mixture was incubated for 60 min, and the absorbance was recorded at 516 and 700 nm using a Shimadzu UV-1700 Pharma Spec (Holm and Halby, Denmark) spectrophotometer. The anthocyanin content was calculated as cyanidin-3-glucoside (cya-glu) equivalents, with a molecular weight of 449.2 and an extinction coefficient of 26,900. Procyanidin (OPC) was determined by the vanillin assay method [24] with an epicatechin standard in the range of 0–120 μg/mL (R_2_ = 0.9996). Briefly, 1 mL of five-fold-diluted juice was mixed with 5 mL of a vanillin aldehyde solution (5 g/100 mL), then 3 mL of hydrochloric acid was added to the mixture. The mixed solution was placed in the dark and allowed to react for 1 h. The absorbance of the solution was read at 500 nm.

The total phenol and flavonoid contents of the juice were measured using spectrophotometry methods [25]. The total phenolic content (TPC) was quantified at 765 nm using a gallic acid standard (R_2_ = 0.9994). The total flavonoid content (TFC) was quantified at 510 nm using a rutin standard (R_2_ = 0.9996).

The DPPH and ABTS radical scavenging capacities of the extracts were determined according to the method of Dudonne et al. [26]. DPPH and ABTS+• radicals exhibit absorption maxima at 515 nm and 734 nm, respectively, that disappear upon reduction by an antioxidant compound. The radical scavenging activity of the extracts was calculated using a Trolox standard.

### 4.6. Physicochemical Parameters

During storage, the total soluble solids was measured using a refractometer (LH-B55, Luheng, Inc., Qingdao, China). The pH was measured using a pH meter (Orion 5-star, Thermo Electron Corporation, Lexington, KY, USA). The color properties were determined using a CR-300 Chroma Meter (Konica Minolta Sensing, Inc., Tokyo, Japan). The total color difference (ΔE) was calculated based on the CIE L* (lightness), a* (redness), and b* (yellowness) values.

## 5. Conclusions

TAHHP treatment at 300 MPa/7 min-55 °C/10 min and 400 MPa/7 min-50 °C/6 min can ensure the microbiological safety of aronia juice during storage at 4 °C for 30 days, whereas HHP cannot. After study of the effect of treatment on the physicochemical properties of aronia juice was studied, it was found that TAHHP and HHP caused significant changes in the juice colour, although these weaker than those observed for TP-treated juices. In addition, juices treated with TAHHP and HHP retained more antioxidants and had a higher antioxidant capacity than TP-treated juices. Therefore, TAHHP may be more effective than TP for extending the shelf life of juice during long-term cold storage. Aronia juice exhibited higher retention of both quality characteristics and natural nutritional compounds beneficial to human health after TAHHP treatment than after TP treatment.

As consumer demand foods that are natural, nutritionally excellent, free from chemical preservatives, and microbiologically safe even with an extended shelf life, and as processing at high temperatures lowers the nutritional quality of foods due to the heat lability of many nutrients, TAHHP (thermal-assisted high hydrostatic pressure treatment) has great potential in future industrial food processing.

## Figures and Tables

**Table 1:** Effects of TAHHP processing on the total aerobic counts in aronia juice.

	Control	200 MPa/7 min	300 MPa/7 min	400 MPa/7 min
Control	5.18 ± 0.06	—	—	—
50 °C/2 min	—	2.79 ± 0.02 Aa	1.11 ± 0.08 Ab	ND
50 °C/6 min	—	2.53 ± 0.02 Ba	ND	ND
50 °C/10 min	—	2.26 ± 0.03 Da	ND	ND
55 °C/2 min	—	2.38 ± 0.04 Ca	1.16 ± 0.12 Ab	ND
55 °C/6 min	—	1.51 ± 0.09 Fa	ND	ND
55 °C/10 min	—	1.66 ± 0.1 Ea	ND	ND

All data are presented as the mean ± SD, n = 3 (log CFU/mL). A, B, and C: different letters in the same column indicate statistically significant differences; a, b, and c: different letters in the same row indicate statistically significant differences (*P* < 0.05). —, not analysed; ND, not detectable (<10 CFU/mL).

**Table 2:** Number of total aerobic counts in TAHHP-treated aronia juice during 30 days of storage at 4 °C.

Treatment	Storage Time (Day)			
	0	10	20	30
400 MPa/7 min	ND	ND	ND	4.93 ± 0.05 Ca
300 MPa/7 min 50 °C/6 min	ND	1.62 ± 0.11 Ac	3.68 ± 0.03 Ab	6.79 ± 0.01 Aa
300 MPa/7 min 50 °C/10 min	ND	ND	1.77 ± 0.06 Bb	6.38 ± 0.04 Ba
300 MPa/7 min 55 °C/6 min	ND	ND	ND	1.49 ± 0.08 Da
300 MPa/7 min 55 °C/10 min	ND	ND	ND	ND
400 MPa/7 min 50 °C/6 min	ND	ND	ND	ND

All data are presented as the mean ± SD, n = 3 (log CFU/mL). A, B, and C: different letters in the same column indicate statistically significant differences; a, b, and c: different letters in the same row indicate statistically significant differences (*P* < 0.05). ND, not detectable (<10 CFU/mL).

**Table 3:** Antioxidant properties of aronia juice during 28 days of storage at 4 °C.

Quality Attribute	Treatment	Storage Time (Days)				
		0	7	14	21	28
Anthocyanins	TAHHP-55	148 ± 2.4 Aa	131 ± 1.1 Ab	129 ± 1.4 Ab	116 ± 1.7 Ac	103 ± 1.6 Ad
(μg/mL)	TAHHP-50	147 ± 2.6 Aa	130 ± 1.5 Ab	130 ± 1.4 Ab	116 ± 1.7 Ac	101 ± 1.8 Ad
	HHP	149 ± 2.6 Aa	129 ± 1.4 Ab	131 ± 1.4 Ab	114 ± 1.5 Ac	92 ± 2.5 Bd
	TP	140 ± 1.3 Ba	123 ± 0.7 Bb	121 ± 0.5 Bb	96 ± 0.6 Bc	77 ± 0.6 Cd
Procyanidins	TAHHP-55	169 ± 1.8 Aa	169 ± 1.6 Aa	170 ± 2.2 Aa	155 ± 3.7 Ab	146 ± 2.4 Ac
(μg/mL)	TAHHP-50	168 ± 1.4 Aa	170 ± 1.2 Aa	169 ± 3.3 Aa	155 ± 2.1 Ab	145 ± 2.7 Ac
	HHP	168 ± 1.8 Aab	170 ± 1.5 Aa	166 ± 2.8 Aab	153 ± 3.0 Ac	143 ± 1.8 Ad
	TP	167 ± 3.6 Aa	165 ± 4.5 Aa	164 ± 3.4 Aa	154 ± 4.8 Ab	145 ± 1.2 Ac
Total flavonoids	TAHHP-55	447 ± 3.2 Aa	451 ± 2.4 Aa	451 ± 3.8 Aa	453 ± 2.4 Aa	452 ± 3.9 Aa
(μg/mL)	TAHHP-50	445 ± 2.8 Aa	450 ± 2.0 Aa	449 ± 2.4 Aa	451 ± 2.4 Aa	459 ± 3.5 Aa
	HHP	450 ± 4.0 Aa	450 ± 1.2 Aa	448 ± 3.6 Aa	452 ± 2.4 Aa	452 ± 2.3 Aa
	TP	450 ± 4.9 Aa	448 ± 1.8 Aa	455 ± 2.8 Aa	458 ± 8.5 Aa	456 ± 5.6 Aa
Total phenolics	TAHHP-55	706 ± 7.0 Aa	684 ± 4.4 Ab	681 ± 3.6 Ab	628 ± 6.5 Ac	628 ± 6.5 Ac
(μg/mL)	TAHHP-50	706 ± 5.6 Aa	680 ± 3.6 Ab	681 ± 4.4 Ab	623 ± 3.4 Ac	623 ± 3.4 Ac
	HHP	704 ± 7.3 Aa	688 ± 5.7 Ab	679 ± 3.9 Ab	622 ± 4.3 Ac	622 ± 4.3 Ac
	TP	688 ± 3.9 Ba	679 ± 3.4 Bb	666 ± 3.9 Bc	628 ± 6.6 Ad	628 ± 4.3 Ac
DPPH	TAHHP-55	801 ± 7.2 Aa	676 ± 4.7 Ab	629 ± 2.6 Ac	481 ± 8.3 Ad	475 ± 3.8 Ad
(μg trolox/mL)	TAHHP-50	792 ± 8.6 Aa	677 ± 4.3 Ab	633 ± 6.1 Ac	471 ± 4.5 Ad	477 ± 3.0 Ad
	HHP	795 ± 6.9 Aa	672 ± 6.5 Ab	641 ± 6.3 Ac	484 ± 14.4 Ad	478 ± 6.7 Ad
	TP	751 ± 5.9 Ba	620 ± 5.9 Bb	588 ± 6.6 Bc	466 ± 9.8 Ad	472 ± 6.3 Ad
ABTS+•	TAHHP-55	1873 ± 38 Aa	1511 ± 24 Ab	1394 ± 21 Ac	1134 ± 11 ABd	1051 ± 14.1 Ae
(μg trolox/mL)	TAHHP-50	1884 ± 30 Aa	1514 ± 22 Ab	1377 ± 24 Ac	1137 ± 14 ABd	1046 ± 17.6 Ae
	HHP	1845 ± 31 Aa	1496 ± 35 ABb	1364 ± 21 Ac	1180 ± 15 Ad	1019 ± 17.9 Ae
	TP	1733 ± 25 Ba	1442 ± 25 Bb	1278 ± 28 Bc	1068 ± 88 Bd	1025 ± 2.1 Ad

All data are presented as the mean ± SD, n = 3. For each indicator: A, B: different letters in the same column indicate statistically significant differences; a, b, c and d: different letters in the same row indicate statistically significant differences (*P* < 0.05).

**Table 4:** Physicochemical characteristics of aronia juice during 28 days of storage at 4 °C.

Quality Attribute	Treatment	Storage Time (Days)				
		0	7	14	21	28
Total soluble solids	TAHHP-55	6.2 ± 0.1 Aa	6.1 ± 0.1 Aa	6.0 ± 0.1 Aa	6.0 ± 0.1 Aa	5.9 ± 0.0 Aa
(°Brix)	TAHHP-50	6.1 ± 0.1 Aa	6.0 ± 0.1 Ab	6.0 ± 0.1 Ab	5.9 ± 0.1 Ab	6.0 ± 0.0 Aa
	HHP	6.1 ± 0.1 Aa	6.0 ± 0.1 Ab	6.0 ± 0.1 Ab	5.9 ± 0.1 Ab	5.7 ± 0.1 Aa
	TP	6.2 ± 0.1 Aa	6.0 ± 0.1 Ab	5.9 ± 0.1 Ab	5.8 ± 0.1 Ab	5.7 ± 0.0 Aa
pH	TAHHP-55	3.8 ± 0.0 Aa	3.7 ± 0.0 Ab	3.6 ± 0.0 Ac	3.6 ± 0.1 Ac	3.6 ± 0.0 Bc
	TAHHP-50	3.8 ± 0.0 Aa	3.7 ± 0.0 Ab	3.6 ± 0.0 Ac	3.6 ± 0.0 Ac	3.6 ± 0.0 Bc
	HHP	3.8 ± 0.0 Aa	3.7 ± 0.0 Ab	3.6 ± 0.0 Ac	3.6 ± 0.0 Ac	3.3 ± 0.0 Cd
	TP	3.8 ± 0.0 Aa	3.7 ± 0.0 Ab	3.7 ± 0.0 Ac	3.6 ± 0.0 Ac	3.7 ± 0.0 Aa
ΔE	TAHHP-55	0.5 ± 0.1 Bd	0.7 ± 0.0 Dc	1.5 ± 0.1 Ba	1.0 ± 0.0 Bb	1.6 ± 0.3 Ba
	TAHHP-50	0.4 ± 0.1 Bd	1.2 ± 0.0 Cb	1.0 ± 0.1 Cc	1.0 ± 0.1 Bc	1.5 ± 0.1 Ba
	HHP	0.2 ± 0.1 Cc	1.3 ± 0.1 Bb	1.1 ± 0.1 Cb	1.1 ± 0.0 Bb	3.9 ± 0.2 Aa
	TP	1.4 ± 0.1 Ad	3.4 ± 0.2 Ac	3.5 ± 0.3 Ac	3.5 ± 0.1 Ab	3.9 ± 0.1 Aa

All data are presented as the mean ± SD, n = 3. A, B, C and D: different letters in the same column indicate statistically significant differences; a, b, c and d: different letters in the same row indicate statistically significant differences (*P* < 0.05).
